# Measurement of China’s public health level: compilation and research of an index

**DOI:** 10.1186/s12889-024-18212-7

**Published:** 2024-03-04

**Authors:** Zhengqi Wei, Keke Wei, Yan Li, Lijie Nie, Yizhuang Zhou

**Affiliations:** 1 Guangxi Key Laboratory of Environmental Exposomics and Entire Lifecycle Health, School of Public Health, Guilin Medical University, Guilin, Guangxi 541199 China; 2 Huazhong University of Science and Technology Tongji Medical College, WuHan, 430000 China

**Keywords:** Public health level system, Economic level, Spatial Durbin model

## Abstract

**Background:**

With the development of the economy, public health has become increasingly important. Therefore, it is important to establish a comprehensive and scientific the public health level index (PHL) system to measure public health level as a research priority. The current research has limitations in exploring the PHL system; therefore, the field still lacks a comprehensive indicator system to measure the level of public health. Therefore, this paper aims to develop a multi-level public health index system and utilizes China as a case study to evaluate its public health status. The objective is to offer insights and recommendations for the improvement of public health initiatives in China and other regions.

**Methods:**

Utilizing data from 2011 to 2020, a comprehensive PHL was developed to encompass three vital indices: the Public Health Service Index (PHS), the Public Health Resource Index (PHR), and the Population Health Level Index (PHL). Subsequently, the PHL, PHS, PHR, and PH were meticulously calculated using a comprehensive evaluation method. Amid the current disparity between public health and economic progress, both the spatial Durbin model and the spatial lag model were finally employed to examine the influence of economic level (EL) on PHL, thus affirming the consistent reliability and accuracy of PHS.

**Results:**

Our findings revealed the following: (*i*) the PHL, PHS, and PHR indices show increasing trends in China; (*ii*) both EL and PHL exhibit high-high clustering and low-low clustering states; (*iii*) the PHL in the area has a positive spatial spillover effect on the surrounding area; (*iv*) EL will result in the siphoning effect of PHL; and (*v*) EL can enhance PHL through urbanization, PH, and PHS.

**Conclusions:**

The PHL system constructed in this paper demonstrates multiple levels, pluralism, spatio-temporal comparability, and robustness. It can reflect not only the input and output of public health initiatives but also the interconnectedness and autonomy within the public health system. Therefore, it can be widely utilized in other areas of public health research.

**Supplementary Information:**

The online version contains supplementary material available at 10.1186/s12889-024-18212-7.

## Background

Public health can be defined as a public service aimed at safeguarding and promoting the health of the population [1]. The original purpose of public health is to emphasize the collaborative efforts of the state and society in improving the natural and social environment related to health, preventing and controlling diseases, providing basic medical and health services, raising people's health awareness, and creating a society where everyone has access to health and equal health resources [2]. This concept originated from Winslow's article titled "The Untilled Fields of Public Health" in 1920 and was later endorsed by various countries and the World Health Organization (WHO) [2]. By 2020, the WHO had initiated projects to improve global public health in 194 member countries, and nations worldwide had set goals to enhance public health [3].

As the largest developing country with the world's largest population, China's public health system and level of development share common characteristics with other countries but also have distinct Chinese features based on national conditions, such as a huge population and the equitable distribution of public health resources. Therefore, examining the evolution of China's public health system and its current status can provide valuable insights into the future development of public health in China, as well as in other developing countries and regions. The Chinese government has consistently made efforts to enhance the overall public health of the nation. Several policies have been implemented. In 2012, the government set the goal of enhancing the capacity of primary medical and public health services as part of the Plan and Implementation Plan for Deepening the Reform of the Medical and Health System during the 12th Five-Year Plan Period [4]. In 2016, the "Healthy China 2030" plan was introduced to establish a high-quality medical and health service system, accelerate the reform of the medical and health system, and improve the public health level [4]. In 2022, the government outlined the 14th Five-Year Plan for Health Standardization, emphasizing the establishment of a strong public health system, the promotion of high-quality advancements in medical and health services, and the improvement of the health of priority populations [5]. Simultaneously, China has significantly increased its financial support, with total health expenditure reaching approximately 7.22 trillion Chinese Yuan (CNY) in 2020 [6]. Although economic development has led to a significant increase in public health resources across China's provinces, there is a clear spatial concentration and uneven regional distribution [7]. This suggests that despite the increase in economic development, the availability of public health resources has improved. However, it remains challenging to meet the growing demand for health resources across provinces [8].

Meanwhile, scholars have been continuously exploring objective, comprehensive, and scientific evaluation indicators to measure public health level in order to identify the deficiencies and improve the development process of public health [9]. Currently, the research methods for public health level evaluation systems mainly include the literature review method [10], panel discussions [11] , and the Delphi method [12, 13]. Xiao-xiao et al. established a fundamental PHL system in Shanghai using the Delphi method. The system comprised eight indicators, including the systematic management rate of children aged 0-6 years, the establishment rate of residents' electronic health records, and the rate of early pregnancy registration [13]. Zhang and Wang developed a PHL system consisting of seven aspects and 17 indicators [14]. The TOPSIS model was used to evaluate the public health level and quality in Zhengzhou. The results revealed that the economic level (EL) significantly promoted the public health level. However, after conducting in-depth research at the public health level, the issues within China's public health service gradually became apparent [15]. Disparities in EL have led to a growing imbalance in access to public health services between urban and rural areas [16]. The efficiency of public health services and investment in healthcare resources are significantly higher in regions with higher economic levels compared to less developed regions [17]. Liu et al. utilized the structure-process-outcome research framework and found that high-level economic and public health services were associated with increased utilization rates of health resources [18]. Due to unbalanced economic development, many cities in less developed areas, such as mountainous regions, are experiencing a shortage of healthcare resources. Meanwhile, government financial subsidies are more targeted toward economically developed regions [19].

Although scholars have constructed many evaluation systems for public health levels, these systems suffer from the problem of incomplete indicator selection. This results in the inability of the indices calculated based on these evaluation systems to comprehensively and effectively reflect the diversity and multi-level nature of public health, the input and output efficiency of public health services, and the level and balance of public health development in various regions. Therefore, guided by the ideology of the "Healthy China 2030" plan, and based on reports from the World Health Organization and relevant public health research, this paper constructs a set of interconnected evaluation indicators for the Public Health Level (PHL) that reflects the current development status of public health from various perspectives. This system includes one overall index, three sub-indices, and eight secondary sub-indices, aiming to fully reflect the current development status of public health, providing scholars with instrumental data possessing the aforementioned characteristics, and promoting further research in the direction of public health. Additionally, previous research has found that the economic level (EL) significantly influences PHL and the balance of PHL [16]. To further verify the robustness and accuracy of the indices constructed in this paper, as well as their similarity to other indices describing the PHL of various regions and the phenomena discovered, this paper utilizes the Spatial Durbin Model and lag model to investigate the impact of EL on PHL.

## Methodology

### Data sources

The original data for this article is sourced from the China Health Statistics Yearbook and national and provincial statistical bureaus. At the same time, in order to minimize the variations in PHL caused by differences in population size between regions, this article preprocesses the original data by normalizing it with the regional population.

### PHL system

The prerequisite for scientifically constructing a PHL is to design a comprehensive and accurate PHL system. Based on the pertinent findings of (Xiao-xiao et al., Yang et al., Zhang and Wang) [13, 14, 20] and the method of Feng et al. [21]. this article establishes the guiding principles for constructing the PHL system as follows.

First, the PHL system should provide a comprehensive overview of the definition and characteristics of public health, including the Public Health Services Index (PHS), the Public Health Resources Index (PHR), and the Population Health Level Index (PH). Each index and dimension should represent a specific aspect of public health. Therefore, it is necessary to consider all three of these indices to realistically depict PHL.

Second, the advancement of public health is a continuous process that evolves alongside the development of the economy and society. The public health levels in the same region can vary significantly from year to year. Similarly, differences in EL, economic structures, and government policies within different regions in the same year will lead to varying levels of public health. The PHL should reflect all of these characteristics, enabling comparisons over time (longitudinal dimension) and across different locations (spatial dimension). Therefore, both horizontal and longitudinal comparability should be taken into consideration.

Third, reflecting the diversity and multilevel nature of public health. With the economic development and greater demand for PHS by the people, it becomes increasingly important to assess whether PHS can meet the people’s needs and the efficiency of utilizing PHS. Therefore, in constructing an indicator system to comprehensively depict PHL, it is necessary to not only reflect the fairness, service quality, and population health aspects of PHS but also include the development and utilization of various types of PHR.

Fourth, reflecting the input-output relationship of public health. PHR is limited, so it is crucial to fully utilize them to meet the public health needs of the people. Therefore, the indicator system constructed in this study can further reflect the input-output relationship of public health: PHR as input, PHS as intermediate output, and population health as final output.

Fifth, Reflecting the interconnectedness and independence within the public health system. The systems (indices) within public health should be both independent and interconnected. Therefore, within the indicator system, indices at the same level should be independent, while higher-level and lower-level indices should be able to be calculated through weight values. In summary, sub-indices are extensions of the overall index, and the overall index is a consolidation of sub-indices. This can more clearly and comprehensively demonstrate the interconnectedness and independence within the public health system, as well as the comprehensiveness of this indicator system.

According to these principles, this article aims to develop the PHL system based on the existing literature [22], public health indicators of the WHO [23], and the specific context of public health development in China. The PHL system was constructed using the dimensions of PHS, PHR, and PH. In China, PHS primarily encompasses maternal and child health care, resident health records management, and health service management [24–26]. As a result, the indicators for the PHS in this paper are categorized into three aspects: maternal and child health care, health security (encompassing all types of health information), and health services. Furthermore, this paper also explores additional indicators to comprehensively and accurately portray the state of PHS. Health resources consist of personnel, funds, and facilities [27]. Therefore, the PHR in this paper is divided into three aspects: health workers, health funds, and health facilities. To provide a comprehensive and accurate portrayal of the PHR situation in Chinese provinces, this paper incorporates relevant indicators into the index system, supplementing those from existing literature [27] and the WHO [23]. Healthy individuals are expected to demonstrate a higher level of awareness of disease prevention, lower mortality rates, and reduced incidence of infection and disease [28]. Therefore, this article focuses on two main aspects: disease prevention and health. It aims to explore relevant indicators that reflect these dimensions. Finally, the PHL system consists of three dimensions: PHS, PHR, and PH, with a total of 50 specific indicators across eight sub-dimensions, as illustrated in Table 1.

**Table 1:** Public health indicator system and indicator weights

Target	Criterion	Secondary criterion	Indicators and Weights
Public health level	Public Health Services (47.169)	Maternal and Child Management (12.574)	Health management rate for children under 7 years old (0.864), postpartum visit rate (0.619), prenatal examination rate (1.915), systematic management rate (0.523), newborn visit rate (0.626), and pregnant woman registration rate (1.385)
		Health Security (25.778)	The proportion of urban employees participating in medical insurance (0.977), the dependency ratio of the elderly population (3.716), per capita healthcare expenditure (RMB) (2.468), the dependency ratio of children and adolescents (2.160), the maternity insurance coverage rate (1.498), and the medical insurance coverage rate (1.341)
		Health Services (61.648)	Sick bed workdays (5.422), bed utilization rate (6.710), observation room mortality rate (2.047), emergency mortality rate (2.288), number of health examinations (person times per thousand people) (2.540), annual average number of visits for residents (1.232), daily average number of doctors responsible for diagnosis and treatment (1.298), daily average number of doctors responsible for hospitalization (2.129), average hospital hospitalization day (4.095), and number of diagnosis and treatment person times (person times per thousand people) (1.319)
	Public Health Resources (27.027)	Health worker (44.529)	Number of staff in maternal and child health centers per thousand people (1.112), number of local health supervision centers per thousand people (1.371), number of staff in disease control centers per thousand people (3.367), number of health technical personnel per thousand people (1.400), number of professional (assistant) physicians per thousand people (1.653), number of registered nurses per thousand people (1.857), number of professional public health personnel per thousand people (1.276)
		Health expenditure (31.593)	Per capita total health expenses (CNY) (1.514), per capita net assets of medical and health institutions (CNY) (1.312), proportion of total health expenses to GDP (1.015), per capita medical expenses for outpatient patients (CNY) (1.477), per capita medical expenses for inpatient patients (CNY) (1.881), and government health expenditure ratio (1.341)
		Sanitary facilities (23.878)	Grassroots medical and health institutions per thousand people (0.975), other medical institutions per thousand people (1.633), number of beds in health institutions per thousand people (1.794), professional public health institutions per thousand people (0.889), and proportion of tertiary hospitals (1.164)
	Population Health Level (25.804)	Disease prevention (48.979)	Women's disease examination rate (%) (1.964), premarital medical examination rate (5.258), incidence rate of Class A and B infectious diseases (1.1 million) (1.808), mortality rate of Class A and B infectious diseases (1.1 million) (1.328), number of public health education activities per thousand people (2.282)
		Resident Health (51.021)	Perinatal mortality rate (‰) (1.561), prevalence of low birth weight in children under 5 years old (1.894), resident hospitalization rate (4.145), mortality rate (2.668), maternal mortality rate (1.1 million) (2.898)

### Indicator calculation method

#### Dimensionless processing

Because indicators in various dimensions of public health contain specific characteristic information about different aspects of public health, drawing conclusions solely based on a certain dimension or indicator may be narrow-minded. Therefore, this article references the methods used in relevant literature and combines multiple indicators of public health into a PHL [21]. Similarly, due to the differences in the properties and units of each indicator, it is necessary to standardize them before creating the PHL. Currently, the primary dimensionless methods in the academic community include the range value method and the power function method [29].

To ensure the stability of the index and minimize the impact of extreme values, the logarithmic power function method, as shown in formula (1), is employed.

1 $$d=\frac{{\text{log}}x-{\text{log}}{x}^{l}}{{\text{log}}{x}^{h}-{\text{log}}{x}^{l}}$$

Where ${\text{log}}{x}^{l}$ and ${\text{log}}{x}^{h}$ are respectively the lower and upper limits of the indicator. If the maximum and minimum values of various indicators in different regions (years) are used as upper and lower limits, it will lead to changes in the comparison benchmark of indicators across different regions (years), resulting in horizontal (vertical) incompatibility of indicators. Therefore, to ensure that the level of public health levels in different regions can be simultaneously compared both horizontally and vertically, this article has implemented the following settings: (*i*) positive indicators, with the upper limit ${x}^{h}$ and lower limit ${x}^{l}$ set as the 95th quantile and 5th quantile of the actual indicator data in each region in 2011, respectively; (*ii*) reverse indicators, with the upper limit ${x}^{h}$ and lower limit ${x}^{l}$ set as the 5th quantile and 95th quantile of the actual indicator data for each region in 2011, respectively; (*iii*) to avoid extreme values and smooth the indices, this article applies tail reduction to the data of the base year: data exceeding the upper limit ${x}^{h}$ or failing below the lower limit ${x}^{l}$ are assigned the values ${x}^{h}$ and ${x}^{l}$, respectively [21].

According to the above method, the dimensionless indicators can be calculated, and the dimensionless values of each indicator in the reference year range from 0 to 100. The higher the score, the greater the level of development of the corresponding indicator. And the data for the years after the base year may exceed the range limit of 0-100.

#### Setting of indicator weights

After dimensionless processing of the indicators, it is necessary to determine the weights for synthesizing each indicator. To fully utilize data information and prevent individual indicators from carrying excessive weight, this article integrates the coefficient of variation method and the entropy weight method to determine weights [30]. Then, the two weights were added together and divided by 2 to obtain the final weight, as illustrated in Table 1. Finally, by using dimensionless data and indicator weights, index synthesis can be conducted layer by layer, from the bottom to the top. The composite index was calculated by multiplying the weighted index of each component by its respective weight. The calculated index and composite index of each layer are presented in Tables S1- S4 in the attached manuscript.

### Spatial Durbin model

Anselin proposed a spatial econometric model to examine the spatial interaction and spatial structure of influencing factors [31]. According to the different forms of spatial effects, spatial measurement models are divided into the spatial Durbin model (SDM), spatial lag model (SLM), and spatial error model (SEM). The spatial Durbin model is a general form of the spatial lag model and the spatial error model. It can obtain unbiased estimates of coefficients. It also considers the explanatory variables of spatial lag and their influence on the explained variables, providing a more accurate estimation of the individual spillover effect and spatial spillover effect [31]. At the same time, the spatial effect that influences the relationship between variables is divided into spatial correlation and spatial heterogeneity [32]. Spatial correlation refers to the mutual influence and consistency of the observed values at different locations in space, while spatial heterogeneity refers to the independence of observed values at different locations, with no mutual influence [32].

The spatial Durbin model formula is shown in Equation 2 as follows:

2 $${\text{Y}}= \uprho\mathrm{WY}+\mathrm{X}\upbeta +\mathrm{WX}\updelta+\upvarepsilon$$

Where ${\text{Y}}$ represents the explained variable, which is PHL in this paper. The variables $\uprho$, $\upbeta$, and $\updelta$ represent the corresponding spatial regression coefficient, while ${\text{W}}$ denotes the spatial weight matrix. ${\text{X}}$ represents the explanatory variable for EL and other control variables in this paper. $\mathrm{WX\delta }$ represents the spatial spillover effect affecting ${\text{Y}}$ [33].

In the spatial Durbin model, the spatial weight matrix can represent the impact of regional position on observation values. Therefore, establishing a suitable setting is crucial for developing spatial Durbin models [34]. Currently, there are three primary types of spatial weight matrix configurations as follows:

- Proximity matrix: It is the simplest spatial weight matrix. The weight of the adjacent matrix is set as follows: if region i and region j have a common edge, ${w}_{ij}=1$, otherwise ${w}_{ij}=0$ [33].
- Distance weight matrix: In 1997, Pace proposed using the longitudinal and latitudinal distance between two places as the basis for the distance weight matrix in the spatial weight matrix, as formular (3) [34].

3 $${W}_{2}=\left(\begin{array}{ccc}{w}_{11}& \cdots & {w}_{1n}\\ \vdots & \ddots & \vdots \\ {w}_{n1}& \cdots & {w}_{nn}\end{array}\right), {W}_{ij}=\left\{\begin{array}{c}\frac{1}{{d}_{ij}}, i\ne j\\ 0, i=j\end{array}\right.$$

Where $\frac{1}{{d}_{ij}}$ is the distance between the geographical centers of two regions.

Economic spatial weight matrix: It is established according to social and economic factors. It can determine weights based on indicators such as per capita GDP, capital flow, and per capita disposable income. The expression is given by formula (4) [ 32].

4 $${W}_{3}=\left(\begin{array}{ccc}{w}_{11}& \cdots & {w}_{1n}\\ \vdots & \ddots & \vdots \\ {w}_{n1}& \cdots & {w}_{nn}\end{array}\right), {W}_{ij}=\left\{\begin{array}{c}\frac{1}{|{\overline{Y} }_{i}-{\overline{Y} }_{j}|}, i\ne j\\ 0, i=j\end{array}\right.$$

Where ${\overline{Y} }_{i}$ is the average per capita real GDP of region *i* during the study period.

This article examines the correlation between PHL and the economy under different weights. Calculate the spatial weight matrix using the three methods mentioned above and construct a spatial Durbin model, with the corresponding spatial weight matrices being set to ${W}_{1}$, ${W}_{2}$ and ${W}_{3}$.

### Selection of variables

This article utilizes the Spatial Durbin model and hysteresis model to examine the influence of the Public Health Level index (PHL) on the economic level (EL) in China. Therefore, the previously derived PHL is set as the dependent variable in the spatial Durbin model and lag model. The indicators in the PHL evaluation system avoid the influence of population on PHL. Therefore, when selecting indicators to reflect the regional PHL, it should be able to avoid the impact of population factors on the economy in different regions of China while fully reflecting the regional PHL. Per capita GDP precisely possesses the aforementioned characteristics [35]. Therefore, this paper selects it to reflect the regional PHL and sets it as the main explanatory variable. At the same time, urbanization, PHS, and PH are selected as control variables. Urbanization rate (UR): UR is an important factor reflecting economic and population distribution, and it can affect the regional PHL by influencing the process of urbanization [36]. Therefore, it is indispensable to include it as a control variable when studying the impact of the economy on PHL. PHS and PH are important components in calculating PHL, and they have certain impacts on PHL, and EL may also be influenced by them and have certain impacts on PHL through them. Therefore, it is necessary to control them when studying the impact of the economy on PHL in order to eliminate the influence of the economy on PHL through them.

### Spatial effect test

To examine the spatial spillover effect of economic development on PHL, a spatial model was employed to determine its influencing mechanism, while Moran's I was used to test the spatial autocorrelation of key variables. Global Moran's index and local Moran's index were calculated for EL and PHL using the spatial weight matrices *W*_*1*_, *W*_*2*_, and *W*_*3*_, respectively. Then, to select the optimal model, we used the Lagrange multipliers (LM) test to ascertain the presence of a spatial effect in the model. Additionally, we used the Likelihood ratio (LR) test and Wald test to determine whether the SDM model would degenerate into a SAR or SEM model. The LR time-space effect inspection was finally used to determine the type of space effect [37].

## Results

### PHL trends

Based on the PHL system and index compilation method described above, this article has compiled the PHL for 31 provinces in China. On the basis of the PHL, this article also compiled the PHS, PHR, and PH, as well as their sub-indices for maternal and child management, security, services, funding, facilities, staff, disease prevention, and resident health. The following is an explanation of the development trend and spatial characteristics of the index.

**Fig. 1:**
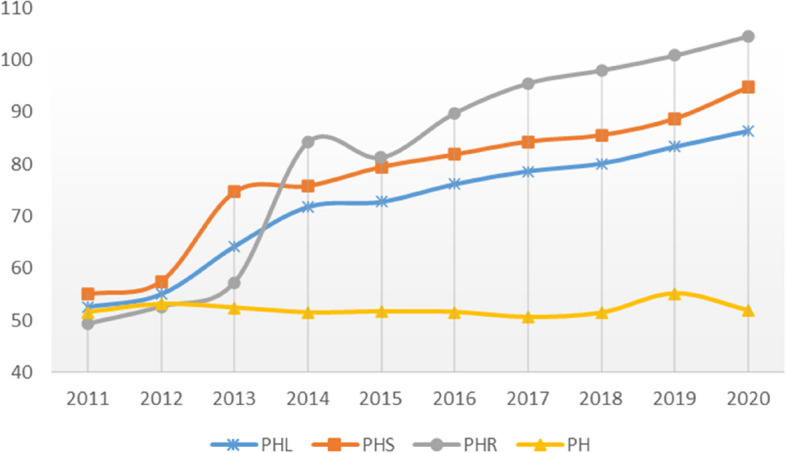
Development trend of average values of various indices

The average PHL of 31 provinces in China increased from 52.592 in 2011 to 86.382 in 2020, representing an annual growth rate of approximately 7.14% (Fig. 1). Among the sub-indices, the PHR experienced the most significant increase, followed by PHS, while PH exhibited a downward trend during the same period. Despite a decline between 2014 and 2015, the PHR remained relatively stable and demonstrated consistent growth. Notably, both PHR and PHS played pivotal roles in the exponential growth of PHL. Conversely, the overall trend of PH showed a gradual decrease as rapid economic development occurred.

**Fig. 2:**
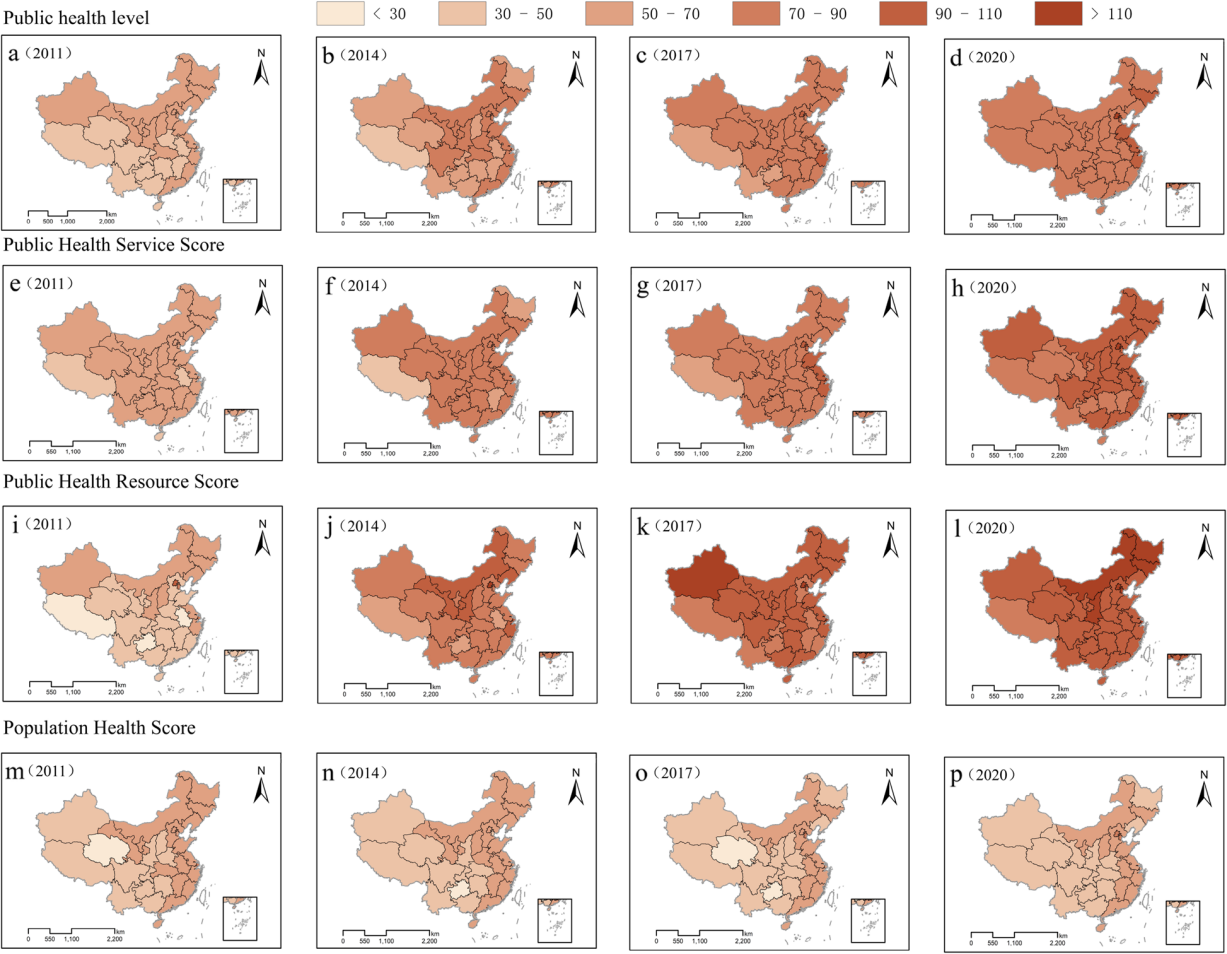
The spatiotemporal variation trend of each index

Figure 2 illustrates the spatiotemporal changes in the PHL, PHS, PHR, and PH of 31 provinces in China. The data reveals an overall upward trend and rapid development in these indicators across the provinces. Specifically, the PHL in each province has increased from less than 70 in 2011 to over 70 in 2020 (Fig. 2a-d). Additionally, the PHS and PHR have increased by nearly two orders of magnitude in almost every province over the past decade (Fig. 2e-l), with several provinces reaching PHR indices exceeding 110 in 2020 (Fig. 2l). However, it is evident that the PH has shown minimal improvement over the past decade (Fig. 2m-p), with certain provinces such as Liaoning, Heilongjiang, and Anhui exhibiting a downward trend. Moreover, the evolution and fluctuations in these indicators underscore an imbalance in China's public health advancement, especially in relation to PHS and PHR. This observation is consistent with the findings of Su et al., who noted that regions with more developed economies receive more corresponding support [19].

### The spatial effect of EL on PHL

#### Spatial autocorrelation test

To examine the spatial spillover effect of EL on PHL, this study uses a spatial model to clarify its impact mechanism and applies Moran's I to evaluate the spatial autocorrelation of core variables. The global Moran's I index for EL and PHL is presented in Table 2, utilizing spatial weight matrices *W*_*1*_, *W*_*2*_, and *W*_*3*_. Across the three spatial weight matrices, the Moran's I index values for EL and PHL are consistently positive. Furthermore, upon integrating Figures S1 and S2 from the Supplementary Materials, it becomes apparent that there is a positive spatial autocorrelation between EL and PHL, indicating the presence of high-high and low-low clustering. This indicates that the EL and PHL of neighboring provinces in China tend to cluster spatially. Furthermore, the spatial correlation between EL and PHL demonstrates a decreasing and U-shaped trend, respectively, across the three weight matrices, indicating strong spatial characteristics. This finding is consistent with the observed cross-regional development and cooperation in EL and PHL.

**Table 2:** Global moran index

Year	EL			PHL		
	${W}_{1}$	${W}_{2}$	${W}_{3}$	${W}_{1}$	${W}_{2}$	${W}_{3}$
2011	0.128***	0.365***	0.422***	0.155***	0.368***	0.327***
2012	0.126***	0.361***	0.425***	0.159***	0.370***	0.288***
2013	0.120***	0.350***	0.424***	0.142***	0.348***	0.303***
2014	0.113***	0.336***	0.417***	0.102***	0.259***	0.253***
2015	0.109***	0.324***	0.420***	0.103***	0.254***	0.316***
2016	0.103***	0.311***	0.401***	0.117***	0.313***	0.318***
2017	0.100***	0.307***	0.390***	0.127***	0.346***	0.338***
2018	0.097***	0.304***	0.378***	0.134***	0.346***	0.280***
2019	0.095***	0.298***	0.372***	0.134***	0.346***	0.307***
2020	0.093***	0.294***	0.375***	0.171***	0.430***	0.279***

Below are the significance signs of the test: *P<0.1, **P<0.05, ***P<0.01

#### Identification and verification of spatial panel models

The results of the spatial panel model identification test are presented in Table 3. The LM test values for the three spatial weight matrices were all statistically significant at the 5% level. This suggests that the models exhibited spatial effects, and the selection of spatial measurement models was appropriate. Additionally, the LR test value and Wald test values were significant at the 5% level, indicating that the SDM model could not be downgraded to SAR or SEM models, and that the SDM model was superior. The LR time-space effect inspection rejected the null hypothesis at the 5% level, leading to the selection of the SDM model. This model is a double fixed effects model that incorporates both time and space. Consequently, this study utilizes the double fixed effects of the Spatial Durbin model to analyze the impact of economic development on PHL.

**Table 3:** Verification of space panel model identification

Inspection type		${W}_{1}$		${W}_{2}$		${W}_{3}$	
		Value	*P*-Value	Value	*P*-Value	Value	*P*-Value
LM	Moran‘s I	22.662	0.000	15.051	0.000	12.342	0.000
	LM-lag	430.153	0.000	206.038	0.000	139.646	0.000
	Robust-LM-lag	332.943	0.000	121.551	0.000	80.813	0.000
	LM-error	165.482	0.000	128.426	0.000	94.668	0.000
	Robust-LM-error	68.272	0.000	43.939	0.000	35.835	0.000
LR	LR-SDM/SEM	72.750	0.000	55.890	0.000	30.280	0.000
	LR-SDM/SAR	56.920	0.000	42.650	0.000	30.970	0.000
Wald	Wald-SDM/SEM	25.730	0.000	30.710	0.000	19.620	0.001
	Wald-SDM/SAR	539.840	0.000	483.000	0.000	358.640	0.000
Spatial effect type test	LR-both/ind	32.200	0.001	50.76	0.000	95.660	0.000
	LR-both/time	534.630	0.000	537.93	0.000	605.51	0.000

#### Spatial Durbin model regression

This article employs the spatial Durbin model to input data into equation (2) and presents the regression results in Table 4, specifically in columns (1), (4), and (7). Under various spatial weight matrices, the regression coefficients for EL are significantly positive at the 1% level, suggesting that EL has a positive spatial influence on regional PHL. Similarly, the coefficients of Rho are positive at the 1% significance level, indicating a positive spatial spillover effect of regional PHL on the surrounding area. This may be attributed to the benefits of location, resources, and talent migrating from regions with enhanced PHL to neighboring areas, consequently boosting PHL in those regions [38]. In contrast, the coefficients of the interaction term WxEL are all negative and statistically significant at the 1% level, indicating that the improvement of EL has a negative spatial spillover effect on PHL. This suggests that while the enhancement of EL promotes local PHL, it results in a decrease in PHL in adjacent regions, possibly due to the strong Matthew effect of PHR (public health practitioners, funds, etc.) [39].

**Table 4:** Spatial durbin regression and lagged multiperiod robustness test

variable	${W}_{1}$			${W}_{2}$			${W}_{3}$		
	(1)	(2)	(3)	(4)	(5)	(6)	(7)	(8)	(9)
L1/L2		0.052***	-0.019*		0.081***	0.009***		0.078***	-0.013***
		4.00	-1.65		5.60	0.535		5.58	-0.95
EL	0.064***	0.059***	0.066***	0.064***	0.055***	0.063***	0.110***	0.093***	0.113***
	3.67	3.50	3.75	3.53	3.10	3.41	6.71	6.49	6.39
UR	0.494***	0.486***	0.491***	0.432***	0.414***	0.432***	0.579***	0.548***	0.581***
	7.44	7.36	7.45	7.13	7.21	7.17	9.44	9.74	9.37
PHS	0.483***	0.467***	0.488***	0.499***	0.474***	0.497***	0.488***	0.470***	0.490***
	17.67	15.64	17.37	16.23	15.56	16.12	16.87	15.86	16.53
PH	0.279***	0.273***	0.279***	0.275***	0.268***	0.275***	0.289***	0.283***	0.288***
	17.83	18.83	17.48	16.63	17.27	16.70	19.20	20.98	18.92
WxEL	-0.157***	-0.142***	-0.160***	-0.093***	-0.097***	-0.095***	-0.139***	-0.132***	-0.138***
	-4.36	-3.90	-4.48	-4.18	-4.12	-4.24	-5.16	-5.30	-5.08
direct effect EL	0.035*	0.038**	0.034*	0.050***	0.041**	0.047**	0.096***	0.079***	0.099***
	1.72	2.27	1.72	2.71	2.48	2.59	5.78	5.82	5.65
indirect effect EL	-0.939***	-0.656**	-0.989**	-0.309**	-0.278***	-0.331*	-0.285**	-0.257***	-0.262*
	-2.72	-2.42	-2.38	-2.44	-3.01	-2.22	-2.26	-2.79	-1.84
Total effect EL	-0.903**	-0.618**	-0.955**	-0.259**	-0.237*	-0.284*	-0.189***	-0.179*	-0.163***
	-2.54	-2.23	-2.24	-1.97	-2.55	-1.85	-1.45	-1.94	-1.11
Rho	0.893***	0.859***	0.895***	0.880***	0.817***	0.878***	0.839***	0.779***	0.841***
	35.13	27.10	35.84	34.61	22.97	34.05	25.62	19.36	25.99
N	310	310	310	310	310	310	310	310	310
R2	0.957***	0.983***	0.981***	0.980***	0.986***	0.980***	0.980***	0.986***	0.980***

L1 and L2 represent PHL with one and two lag periods, respectively

Below are the significance signs of the test: *P<0.1, **P<0.05, ***P<0.01

Additionally, the direct and indirect effects of EL indicate a direct promotional effect and an indirect inhibitory effect on PHL, respectively. This implies that the improvement of EL can contribute to the development of regional PHL, while a rise in EL in neighboring regions may reduce PHL in those regions. This observation is in line with the earlier conclusions [17]. Furthermore, since the direct effect has a lesser promoting impact compared to the inhibitory effect of the indirect effect, the overall effect is inhibitory. This suggests that when the EL of a region improves, it has an average inhibitory effect on the PHL of all regions, reflecting the uneven development of PHR, PHS, and PHL in various provinces of China. Control variables indicate that rapid EL has resulted in accelerated urbanization, which in turn necessitates an expansion of public health facilities to improve PHS, thereby contributing to the overall improvement of PHL [40].

To further confirm the stationarity of the spatial Durbin model, this article utilizes a lag 2-period regression approach and presents the regression results in columns (2), (3), (5), (6), (8), and (9) of Table 4. The coefficients of the first and second lagging periods for PHL are significantly positive at the 1% level, indicating that the development of previous periods of PHL significantly promotes subsequent period development. The coefficients of EL are also significantly positive in both the lagged and non-lagged periods, indicating strong stationarity in the model. Furthermore, the spatial correlation coefficient Rho is significantly positive when lagged for a period, indicating that the impact of the economy on PHL remains consistent over time.

## Discussion

### PHL system

The PHL system constructed in this paper differs from previous studies in its emphasis. Most of the indicator systems in previous studies have focused on reflecting a specific aspect or perspective of public health. For instance, Xiao-xiao et al. assessed, ranked, and graded the basic PHL of community health service centers [13], whereas Zhang and Wang concentrated on evaluating and ranking the quality of basic PHS [14]. Chen et al. examined the developmental status of PHL and PHR in China from the perspective of economy and finance [7]. Guo et al. developed an indicator system using the PHS that was accessible to both urban and rural residents. This system was used to investigate the impact of the economy on PHL and the rate of resource utilization [16]. Li et al. developed an indicator system to assess performance and PHL from a systemic perspective, aiming to enhance the public health system [15]. In a similar vein, Liu Shuo conducted research on the service capacity of public health institutions, employing the structure-process-outcome framework as the basis for their study [18]. The PHL system constructed in this paper is an "index family" that comprises multiple levels and indices, enabling it to reflect the PHL of various regions from diverse perspectives. It can fully capture the temporal and spatial characteristics of PHL, the diversity and multilevel nature of public health, the input and output of public health, and the interconnectedness and independence within the public health system. However, there are limitations to the application of the index system constructed in this paper. When applied to the evaluation of more specific administrative regions such as cities and counties, an excessive number of indicators requires additional financial support for the PHL. As a result, the index system constructed in this paper can only be applied at the provincial level. Nevertheless, due to the characteristics of the calculation method used in this paper’s index system, it can be quickly updated after new index data is added. Therefore, this paper aims to apply the index system to more specific administrative regions and continue to update this "index family".

### Development trend of PHL, PHS, PHR and PH

During the study period, PHL, PHS, and PHR showed a rapid development trend, which may be closely associated with the government's efforts to strengthen the construction of the public health system and increase investment in related facilities [41]. Additionally, the state's proposed incentive policies, development goals, and financial support during this period also contributed to this trend [4, 6]. However, PH showed the opposite trend, remaining unchanged or even slightly decreased. This phenomenon is closely related to the rapid pace of Chinese society, the increasingly intense competition, and the incomplete implementation of work systems. These factors contribute to irregular lifestyles, hindering residents from effectively resting and relaxing their bodies and minds [42, 43]. In response, the government can improve the implementation of the "8-hour working day" and enhance the work system to promote people's health. Moreover, this trend may also have a significant causal relationship with air pollution, food additives, light pollution, and chemical pollution resulting from rapid economic development [42]. The government can tackle these issues by improving food safety and addressing environmental pollution. In terms of food safety, the government should consider aligning the dosage of food additives in food with the standards of European and American countries. Additionally, the government should enhance penalties for enterprises and businesses found to have food safety issues. In addressing environmental concerns, the government should prioritize promoting of new energy vehicles, upgrading the waste/wastewater treatment and disposal systems of industrial enterprises, and strengthening the enforcement of penalties for excessive pollutant emissions. Furthermore, these trends may also be strongly linked to public health emergencies, such as COVID-19 [44, 45]. Therefore, national and regional governments should prioritize the development of a strong and resilient medical and healthcare system. This system should be able to quickly detect, report, and monitor public health emergencies. Additionally, it should provide accurate, comprehensive, and timely information to help the public understand the risks and responses to public health emergencies. Furthermore, a robust healthcare system can strategically allocate medical resources, including personnel, equipment, and medication, to ensure that patients receive prompt medical care and minimize the impact of the epidemic on public health. It can also improve public health awareness and promote behavioral change through educational and awareness campaigns aimed at reducing the spread of the disease [46].

### The impact of economy on PHL

EL has a significant impact on PHL, as numerous studies have demonstrated its effects on various aspects such as quality, resource allocation, fiscal subsidies, and performance [13–16, 19]. This paper also confirms similar findings, emphasizing the contributions of both EL and PHL to cross-regional development and cooperation. While EL can enhance local PHL, it also has a "siphon effect" on the surrounding area, as observed in previous studies [13, 14]. This phenomenon inevitably leads to an unbalanced distribution and utilization of PHR among regions, resulting in significant inequality in PHS and healthcare quality across different regions [18]. Urbanization accelerates the demand for and quantity of public health facilities and services, thereby increasing PHL. However, mindless urbanization may result in an imbalance between the supply and demand of PHR, leading to the opposite effect [40]. To ensure the balanced promotion of PHL in various regions, it is essential to maximize its economic impact. National and regional governments can make efforts in terms of financing, healthcare resources, subsidies, and human resources. The government should allocate public health transfer funds specifically to economically underdeveloped areas. This will encourage local governments to develop public health initiatives and improve public health welfare. Additionally, creating a support system between developed and less developed regions can help achieve a more equitable distribution of PHR. Regulating PHR based on the population structure and demand in each region can improve utilization and reduce imbalances. Moreover, offering financial subsidies to incentivize the relocation of PHR to regions with low PHL and establishing a comprehensive talent training mechanism can help alleviate the disparity in medical and health service expertise.

## Conclusion

Here, we have developed a PHL system with multiple levels, pluralism, spatio-temporal comparability, and robustness. This system not only reflects the input-output of public health initiatives but also the interconnectedness and interdependence within the public health system. The accuracy and robustness of the model were demonstrated by using the spatial Durbin model and spatial lag model to compare the similarity of EL's influence on PHL as observed in previous studies. We made several discoveries during the verification process. First, the levels of PHL, PHS, and PHR showed an increasing trend in China during the study period, while PH experienced a slight decrease. Second, EL and PHL showed positive spatial autocorrelation, indicating high-high and low-low clustering. EL has a positive spatial impact on PHL in its region. Third, PHL demonstrated a spatial spillover effect on the surrounding area. Fourth, because of the clear differences in the development of PHR and PHS between regions, EL will encourage the transfer of resources from regions with surplus public health resources to those with deficits (siphon effect of PHL). Fifth, enhancing urbanization, PH, and PHS can enhance EL and consequently facilitate the development of PHL. This study offers references and recommendations for the future advancement of public health in China and other developing countries and regions.

### Supplementary Information

**Supplementary Material 1.** 

## Data Availability

The datasets used and/or analysed during the current study available from the corresponding author on reasonable request.
