# Supplementary material for: Measurement of China’s public health level: compilation and research of an index
Source: BMC Public Health. 2024 Mar 4;24:686. doi: 10.1186/s12889-024-18212-7 (PMC10913443; doi:10.1186/s12889-024-18212-7)
Supplement: Supplementary file 1 — Supplementary Material 1. [file 12889_2024_18212_MOESM1_ESM.docx]

Measurement of China's Public Health Level: Compilation and Research of an Index


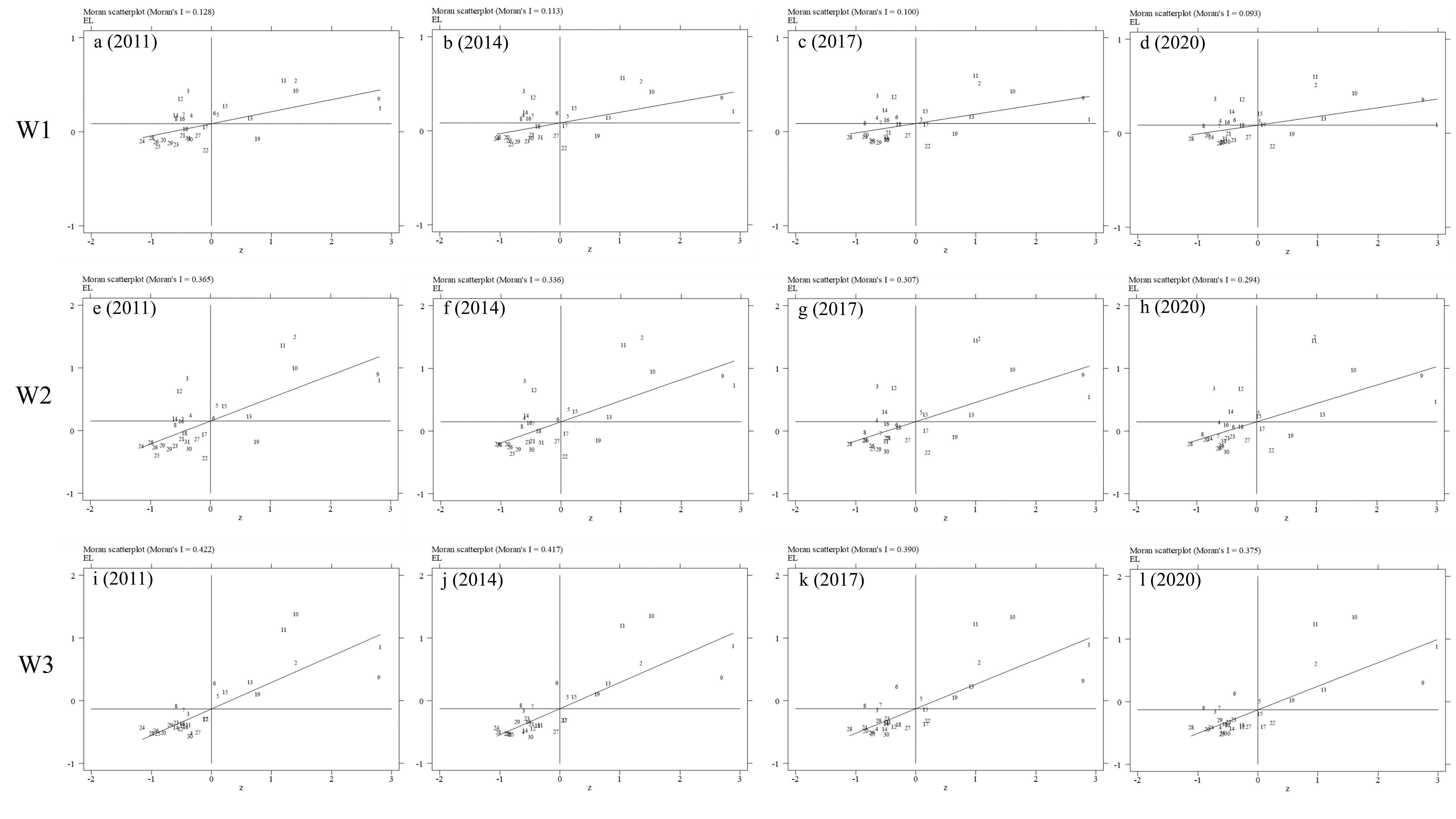
Figure S 1 Local Moran exponent scatter plot of EL


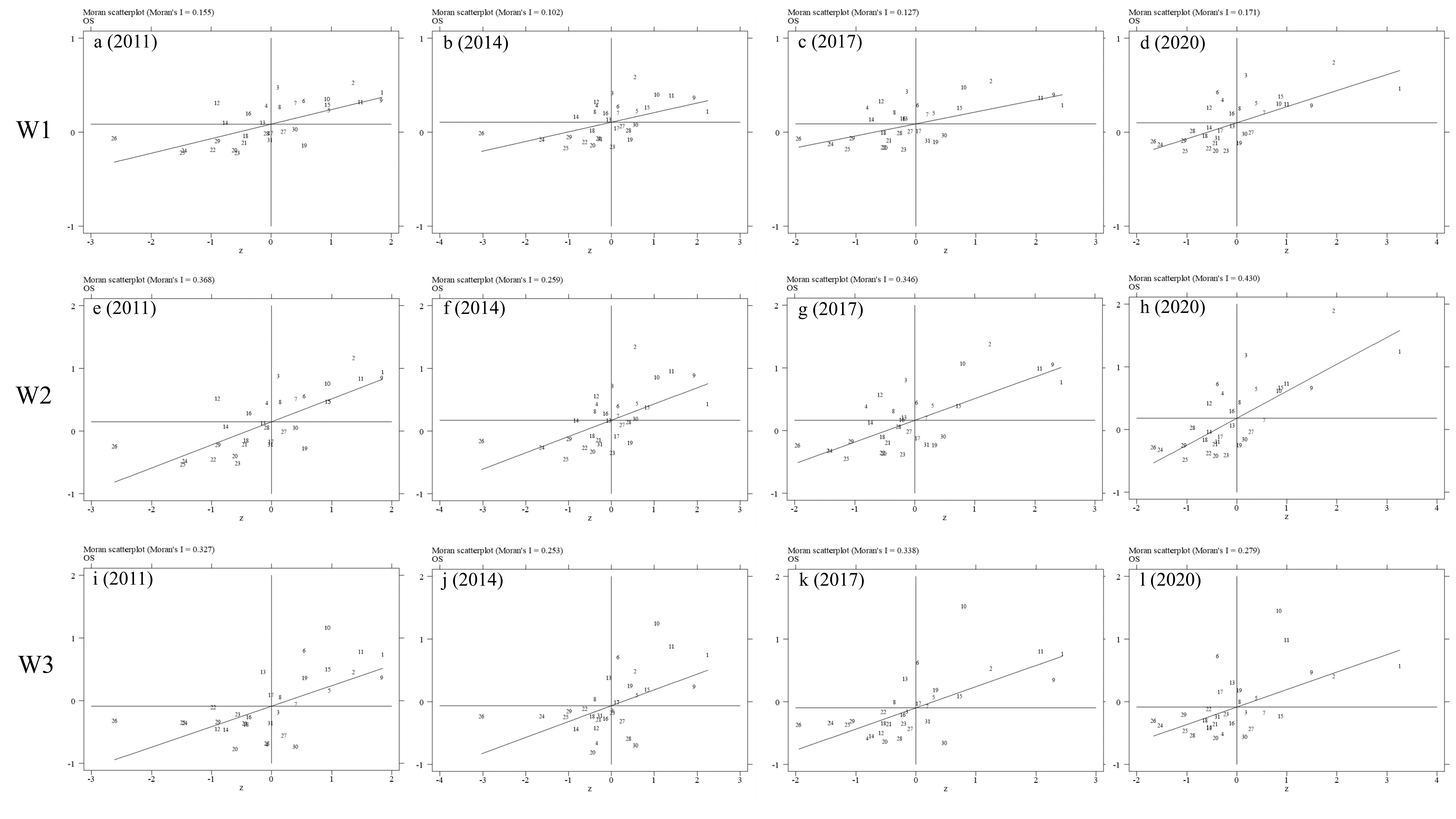


Figure S 2 Local Moran exponent scatter plot of OS

Table S 1 Public Health Level Index by Province from 2011 to 2020

|  | 2011 | 2012 | 2013 | 2014 | 2015 | 2016 | 2017 | 2018 | 2019 | 2020 |
| --- | --- | --- | --- | --- | --- | --- | --- | --- | --- | --- |
| Beijing | 66.037 | 72.527 | 82.481 | 89.008 | 92.219 | 96.537 | 97.745 | 97.520 | 104.952 | 110.816 |
| Tianjin | 62.506 | 65.601 | 74.486 | 76.059 | 73.985 | 86.137 | 88.477 | 90.516 | 92.655 | 100.994 |
| Hebei | 53.440 | 54.892 | 63.380 | 72.051 | 73.079 | 74.239 | 77.530 | 81.120 | 85.303 | 87.755 |
| Shanxi | 52.053 | 59.693 | 62.417 | 69.201 | 69.459 | 71.086 | 72.339 | 75.822 | 77.733 | 84.252 |
| Inner Mongolia | 59.610 | 60.099 | 68.787 | 76.379 | 76.996 | 78.837 | 81.018 | 81.242 | 85.029 | 89.304 |
| Liaoning | 56.605 | 59.870 | 66.968 | 73.014 | 73.839 | 76.990 | 78.915 | 78.441 | 80.336 | 83.516 |
| Jilin | 55.559 | 58.286 | 68.943 | 73.037 | 75.998 | 78.718 | 80.177 | 82.458 | 85.561 | 90.472 |
| Heilongjiang | 53.649 | 55.072 | 63.402 | 68.880 | 69.793 | 73.169 | 75.865 | 76.807 | 80.285 | 86.784 |
| Shanghai | 65.944 | 70.477 | 80.798 | 86.603 | 90.409 | 93.782 | 96.637 | 96.945 | 98.524 | 97.615 |
| Jiangsu | 59.371 | 61.959 | 71.871 | 79.968 | 81.416 | 84.379 | 84.937 | 85.988 | 90.478 | 92.736 |
| Zhejiang | 63.424 | 66.154 | 74.427 | 82.613 | 84.429 | 88.012 | 94.960 | 91.314 | 95.195 | 93.886 |
| Anhui | 46.070 | 50.262 | 60.855 | 69.156 | 69.302 | 72.096 | 74.125 | 76.839 | 80.610 | 82.238 |
| Fujian | 51.611 | 54.002 | 61.974 | 71.409 | 73.889 | 75.975 | 77.263 | 78.102 | 81.645 | 85.720 |
| Jiangxi | 47.036 | 49.067 | 56.831 | 65.514 | 66.331 | 69.166 | 72.875 | 74.859 | 78.285 | 82.252 |
| Shandong | 59.459 | 62.165 | 71.244 | 78.216 | 78.272 | 81.467 | 84.425 | 88.275 | 91.040 | 92.970 |
| Henan | 49.876 | 54.579 | 63.848 | 70.816 | 71.819 | 75.635 | 76.988 | 78.930 | 82.535 | 85.639 |
| Hubei | 52.560 | 54.374 | 63.231 | 72.816 | 75.479 | 77.558 | 79.046 | 79.885 | 82.672 | 83.921 |
| Hunan | 49.520 | 51.478 | 60.776 | 68.429 | 69.083 | 73.277 | 74.452 | 76.459 | 79.780 | 81.647 |
| Guangdong | 56.623 | 59.606 | 68.907 | 75.200 | 76.220 | 79.894 | 81.254 | 82.605 | 85.754 | 86.754 |
| Guangxi | 48.154 | 49.521 | 59.285 | 68.464 | 70.101 | 73.871 | 74.604 | 77.945 | 79.628 | 83.231 |
| Hainan | 49.380 | 51.623 | 59.910 | 69.553 | 71.298 | 73.089 | 75.163 | 75.480 | 77.594 | 83.148 |
| Chongqing | 45.529 | 49.190 | 59.894 | 67.126 | 69.800 | 72.567 | 74.449 | 76.283 | 80.092 | 82.151 |
| Sichuan | 48.521 | 51.184 | 62.333 | 72.008 | 71.524 | 74.862 | 77.104 | 79.814 | 83.210 | 84.817 |
| Guizhou | 42.075 | 42.827 | 51.804 | 59.384 | 58.773 | 63.856 | 67.551 | 68.610 | 72.451 | 74.942 |
| Yunnan | 41.846 | 44.527 | 55.366 | 63.709 | 64.297 | 68.036 | 69.741 | 72.413 | 75.233 | 78.666 |
| Tibet | 33.577 | 33.579 | 45.150 | 48.551 | 55.840 | 56.989 | 63.432 | 64.462 | 68.934 | 73.930 |
| Shaanxi | 54.141 | 57.678 | 66.546 | 73.821 | 74.126 | 76.787 | 77.966 | 81.485 | 85.265 | 88.526 |
| Gansu | 52.052 | 54.534 | 64.212 | 74.965 | 69.194 | 74.881 | 76.544 | 76.138 | 77.886 | 79.714 |
| Qinghai | 46.079 | 41.453 | 51.338 | 64.243 | 63.326 | 65.235 | 70.342 | 74.723 | 75.799 | 78.404 |
| Ningxia | 55.548 | 56.291 | 67.079 | 76.172 | 75.563 | 80.365 | 82.415 | 83.176 | 85.732 | 87.582 |
| Xinjiang | 52.513 | 54.638 | 62.963 | 69.778 | 70.234 | 74.360 | 80.254 | 79.429 | 84.184 | 83.469 |

Table S 2 Public Health Service Index by Province from 2011 to 2020

|  | 2011 | 2012 | 2013 | 2014 | 2015 | 2016 | 2017 | 2018 | 2019 | 2020 |
| --- | --- | --- | --- | --- | --- | --- | --- | --- | --- | --- |
| Beijing | 57.572 | 61.628 | 81.614 | 85.747 | 92.415 | 97.901 | 100.613 | 99.780 | 104.227 | 112.567 |
| Tianjin | 60.702 | 65.751 | 82.818 | 82.549 | 82.627 | 88.818 | 91.193 | 95.259 | 95.984 | 109.734 |
| Hebei | 63.694 | 63.877 | 78.295 | 79.456 | 82.729 | 84.972 | 88.400 | 90.971 | 94.926 | 97.664 |
| Shanxi | 52.819 | 63.298 | 68.619 | 70.060 | 73.597 | 74.347 | 74.563 | 77.344 | 80.811 | 90.047 |
| Inner Mongolia | 57.520 | 58.254 | 74.951 | 77.589 | 78.149 | 79.961 | 81.645 | 80.890 | 85.038 | 94.702 |
| Liaoning | 52.373 | 56.787 | 73.024 | 74.051 | 77.768 | 81.469 | 84.309 | 86.577 | 90.585 | 96.300 |
| Jilin | 53.785 | 52.368 | 73.665 | 73.436 | 79.555 | 78.677 | 82.099 | 82.645 | 86.901 | 94.648 |
| Heilongjiang | 52.301 | 52.914 | 67.843 | 68.222 | 72.483 | 75.612 | 78.901 | 79.207 | 81.977 | 93.704 |
| Shanghai | 56.615 | 63.324 | 84.721 | 86.591 | 95.927 | 98.281 | 101.607 | 102.585 | 105.439 | 106.028 |
| Jiangsu | 60.672 | 63.958 | 82.714 | 83.034 | 88.111 | 90.028 | 91.295 | 92.028 | 96.759 | 101.294 |
| Zhejiang | 63.189 | 65.103 | 82.305 | 84.287 | 90.964 | 92.761 | 106.071 | 97.220 | 101.777 | 102.271 |
| Anhui | 45.553 | 52.572 | 74.571 | 73.734 | 77.761 | 79.614 | 81.975 | 84.989 | 89.277 | 93.150 |
| Fujian | 53.598 | 58.665 | 72.750 | 73.059 | 79.492 | 80.441 | 81.032 | 81.749 | 85.345 | 92.365 |
| Jiangxi | 51.774 | 52.561 | 68.322 | 69.768 | 72.565 | 73.745 | 76.999 | 78.281 | 81.493 | 88.796 |
| Shandong | 65.879 | 68.812 | 84.803 | 85.136 | 87.113 | 90.838 | 94.851 | 99.865 | 102.511 | 104.785 |
| Henan | 56.039 | 57.540 | 75.270 | 74.181 | 77.663 | 82.622 | 82.158 | 85.228 | 88.506 | 95.018 |
| Hubei | 55.580 | 57.423 | 75.188 | 76.788 | 80.265 | 82.726 | 83.791 | 85.441 | 87.779 | 94.681 |
| Hunan | 52.217 | 53.520 | 71.381 | 73.526 | 75.421 | 80.385 | 79.918 | 82.700 | 85.762 | 89.481 |
| Guangdong | 60.762 | 65.136 | 81.827 | 81.261 | 82.837 | 84.737 | 85.920 | 87.251 | 89.162 | 94.710 |
| Guangxi | 55.579 | 57.039 | 74.492 | 75.694 | 78.973 | 80.088 | 79.831 | 83.648 | 85.220 | 92.334 |
| Hainan | 48.851 | 54.417 | 69.308 | 72.810 | 74.215 | 74.679 | 76.815 | 77.810 | 80.755 | 86.796 |
| Chongqing | 54.104 | 56.237 | 78.837 | 80.959 | 84.687 | 86.276 | 86.931 | 88.106 | 91.621 | 95.675 |
| Sichuan | 55.650 | 57.238 | 78.815 | 82.304 | 82.376 | 83.843 | 84.673 | 88.131 | 91.601 | 97.003 |
| Guizhou | 53.321 | 54.145 | 70.973 | 72.090 | 72.575 | 76.623 | 78.832 | 81.659 | 83.746 | 85.188 |
| Yunnan | 51.207 | 52.812 | 69.438 | 70.339 | 73.109 | 74.165 | 74.892 | 75.284 | 78.315 | 85.503 |
| Tibet | 38.935 | 37.265 | 52.129 | 48.491 | 61.932 | 59.140 | 66.197 | 64.094 | 70.893 | 84.766 |
| Shaanxi | 55.951 | 58.049 | 75.004 | 77.917 | 80.859 | 81.814 | 83.106 | 85.113 | 88.725 | 96.819 |
| Gansu | 57.360 | 58.671 | 75.364 | 73.872 | 74.090 | 82.660 | 85.940 | 84.841 | 83.841 | 88.900 |
| Qinghai | 54.163 | 51.595 | 67.203 | 71.341 | 73.888 | 76.122 | 79.443 | 80.037 | 81.368 | 84.585 |
| Ningxia | 54.946 | 56.391 | 75.626 | 76.980 | 78.050 | 82.782 | 85.295 | 87.250 | 87.895 | 93.896 |
| Xinjiang | 53.081 | 55.596 | 72.647 | 74.782 | 81.210 | 81.153 | 85.458 | 86.848 | 92.667 | 95.650 |

Table S 3 Public Health Resource Index by Province from 2011 to 2020

|  | 2011 | 2012 | 2013 | 2014 | 2015 | 2016 | 2017 | 2018 | 2019 | 2020 |
| --- | --- | --- | --- | --- | --- | --- | --- | --- | --- | --- |
| Beijing | 91.971 | 97.911 | 104.498 | 123.780 | 119.801 | 128.633 | 131.393 | 133.803 | 136.905 | 138.084 |
| Tianjin | 75.809 | 75.184 | 77.429 | 89.759 | 84.953 | 92.827 | 97.613 | 100.115 | 103.644 | 113.160 |
| Hebei | 37.981 | 43.708 | 50.093 | 77.149 | 74.065 | 78.150 | 83.303 | 87.696 | 91.673 | 96.105 |
| Shanxi | 53.938 | 58.305 | 59.843 | 82.988 | 79.219 | 87.764 | 91.817 | 96.392 | 96.815 | 103.098 |
| Inner Mongolia | 59.230 | 62.051 | 67.557 | 93.460 | 92.075 | 98.560 | 103.862 | 106.487 | 109.473 | 114.893 |
| Liaoning | 64.536 | 68.643 | 70.031 | 90.871 | 87.490 | 95.530 | 99.344 | 94.759 | 92.651 | 98.913 |
| Jilin | 60.425 | 65.924 | 67.463 | 89.910 | 87.194 | 95.996 | 99.075 | 103.559 | 104.981 | 112.147 |
| Heilongjiang | 58.583 | 62.894 | 66.630 | 87.695 | 84.862 | 92.017 | 96.733 | 98.367 | 99.656 | 110.775 |
| Shanghai | 77.662 | 78.379 | 86.191 | 102.924 | 100.871 | 109.840 | 114.654 | 116.084 | 117.779 | 119.144 |
| Jiangsu | 50.323 | 51.688 | 56.626 | 85.485 | 83.580 | 91.893 | 96.464 | 99.691 | 103.616 | 106.060 |
| Zhejiang | 61.905 | 64.020 | 66.591 | 94.173 | 91.425 | 101.138 | 104.873 | 106.322 | 108.578 | 106.851 |
| Anhui | 29.584 | 32.272 | 34.111 | 65.234 | 61.122 | 69.445 | 74.746 | 77.427 | 80.490 | 90.611 |
| Fujian | 40.998 | 42.271 | 46.993 | 79.291 | 75.720 | 83.696 | 88.390 | 91.738 | 94.998 | 98.285 |
| Jiangxi | 31.505 | 38.785 | 41.476 | 71.284 | 69.898 | 77.643 | 83.976 | 86.896 | 91.999 | 98.499 |
| Shandong | 45.816 | 51.383 | 56.430 | 83.776 | 80.876 | 89.189 | 93.473 | 97.939 | 100.880 | 104.283 |
| Henan | 43.882 | 50.736 | 55.065 | 84.106 | 81.400 | 88.882 | 94.914 | 97.912 | 101.036 | 103.457 |
| Hubei | 49.230 | 52.815 | 55.768 | 88.250 | 88.011 | 95.940 | 100.408 | 102.206 | 104.123 | 107.222 |
| Hunan | 46.089 | 49.810 | 53.482 | 79.890 | 77.725 | 86.252 | 92.357 | 95.225 | 101.602 | 104.881 |
| Guangdong | 48.039 | 50.911 | 57.686 | 81.209 | 77.812 | 88.465 | 93.112 | 96.802 | 100.003 | 99.548 |
| Guangxi | 40.540 | 46.259 | 49.732 | 79.162 | 76.505 | 86.203 | 92.374 | 96.630 | 99.164 | 103.421 |
| Hainan | 47.612 | 48.726 | 55.670 | 82.052 | 83.268 | 90.795 | 96.276 | 95.154 | 97.662 | 100.614 |
| Chongqing | 37.739 | 43.563 | 46.233 | 71.056 | 72.435 | 82.512 | 89.070 | 93.286 | 97.085 | 99.368 |
| Sichuan | 40.909 | 45.829 | 51.603 | 80.509 | 76.846 | 87.213 | 93.491 | 96.888 | 101.979 | 105.669 |
| Guizhou | 28.258 | 33.100 | 39.317 | 68.448 | 65.686 | 77.099 | 86.279 | 90.461 | 94.945 | 96.568 |
| Yunnan | 33.423 | 36.598 | 40.561 | 72.518 | 71.331 | 82.229 | 88.765 | 91.404 | 96.218 | 102.929 |
| Tibet | 19.083 | 16.267 | 28.599 | 57.571 | 58.890 | 67.215 | 77.014 | 81.107 | 85.452 | 85.687 |
| Shaanxi | 56.684 | 62.460 | 67.758 | 90.392 | 87.128 | 95.786 | 97.298 | 105.195 | 108.309 | 112.242 |
| Gansu | 41.963 | 46.827 | 51.636 | 97.233 | 78.733 | 86.330 | 92.065 | 93.484 | 98.546 | 99.288 |
| Qinghai | 48.640 | 46.426 | 54.132 | 83.254 | 81.774 | 88.697 | 97.119 | 99.821 | 101.931 | 103.644 |
| Ningxia | 48.851 | 49.561 | 56.584 | 87.805 | 84.263 | 96.151 | 100.954 | 102.688 | 104.762 | 107.109 |
| Xinjiang | 57.442 | 58.122 | 60.981 | 89.121 | 83.107 | 91.448 | 110.038 | 102.464 | 100.440 | 99.056 |

Table S 4 Population Health Index by Province from 2011 to 2020

|  | 2011 | 2012 | 2013 | 2014 | 2015 | 2016 | 2017 | 2018 | 2019 | 2020 |
| --- | --- | --- | --- | --- | --- | --- | --- | --- | --- | --- |
| Beijing | 54.348 | 65.864 | 61.007 | 58.552 | 62.975 | 60.428 | 57.265 | 55.389 | 72.811 | 79.057 |
| Tianjin | 51.870 | 55.292 | 56.175 | 49.850 | 46.702 | 74.233 | 73.947 | 71.793 | 75.061 | 72.278 |
| Hebei | 50.889 | 50.184 | 50.036 | 53.176 | 54.410 | 50.523 | 51.615 | 56.225 | 61.041 | 60.898 |
| Shanxi | 48.678 | 54.557 | 53.777 | 53.192 | 51.675 | 47.658 | 47.873 | 51.499 | 52.122 | 53.919 |
| Inner Mongolia | 63.830 | 61.429 | 58.811 | 56.278 | 59.098 | 56.127 | 55.945 | 55.445 | 59.413 | 52.637 |
| Liaoning | 56.034 | 56.321 | 52.690 | 52.418 | 52.362 | 49.386 | 47.658 | 46.478 | 48.704 | 44.022 |
| Jilin | 53.706 | 61.104 | 61.863 | 54.636 | 57.772 | 60.698 | 56.872 | 60.018 | 62.776 | 60.137 |
| Heilongjiang | 50.945 | 50.826 | 51.905 | 50.379 | 49.096 | 48.962 | 48.458 | 49.839 | 56.905 | 49.007 |
| Shanghai | 70.726 | 75.277 | 67.979 | 69.532 | 69.367 | 68.740 | 68.681 | 66.590 | 65.720 | 59.688 |
| Jiangsu | 66.471 | 69.065 | 68.017 | 68.585 | 66.912 | 66.183 | 61.244 | 60.598 | 65.238 | 63.139 |
| Zhejiang | 65.445 | 70.313 | 68.234 | 67.446 | 65.157 | 65.584 | 64.271 | 64.801 | 69.149 | 64.980 |
| Anhui | 64.281 | 64.882 | 63.794 | 64.898 | 62.409 | 61.132 | 59.125 | 61.325 | 64.897 | 53.522 |
| Fujian | 59.098 | 57.766 | 57.967 | 60.139 | 61.731 | 59.728 | 58.722 | 57.157 | 60.899 | 60.414 |
| Jiangxi | 54.641 | 53.451 | 51.908 | 51.698 | 51.200 | 51.919 | 53.711 | 55.999 | 58.058 | 53.274 |
| Shandong | 62.014 | 61.308 | 61.975 | 59.747 | 59.386 | 56.253 | 55.892 | 56.967 | 59.767 | 59.526 |
| Henan | 44.890 | 53.192 | 52.171 | 50.745 | 51.101 | 48.991 | 48.766 | 47.536 | 52.244 | 49.834 |
| Hubei | 50.526 | 50.434 | 49.191 | 49.393 | 53.608 | 48.859 | 48.001 | 46.351 | 50.870 | 39.848 |
| Hunan | 48.184 | 49.494 | 49.030 | 47.108 | 48.446 | 46.698 | 45.710 | 45.397 | 45.990 | 42.995 |
| Guangdong | 58.051 | 58.607 | 57.043 | 57.827 | 62.457 | 62.065 | 60.304 | 59.243 | 64.600 | 58.812 |
| Guangxi | 42.558 | 39.196 | 41.494 | 44.045 | 47.179 | 49.594 | 46.437 | 47.952 | 48.948 | 45.446 |
| Hainan | 52.202 | 49.551 | 47.175 | 50.509 | 53.431 | 51.638 | 50.034 | 50.617 | 50.798 | 58.189 |
| Chongqing | 38.015 | 42.204 | 39.577 | 37.724 | 39.829 | 37.092 | 36.321 | 36.863 | 41.219 | 39.400 |
| Sichuan | 43.460 | 45.728 | 43.444 | 44.284 | 46.115 | 45.511 | 46.108 | 46.728 | 48.216 | 40.702 |
| Guizhou | 35.990 | 32.325 | 29.841 | 26.666 | 26.303 | 26.651 | 27.314 | 21.872 | 28.247 | 33.562 |
| Yunnan | 33.559 | 37.687 | 45.152 | 42.365 | 40.823 | 41.969 | 40.402 | 47.276 | 47.621 | 40.754 |
| Tibet | 38.962 | 44.976 | 49.730 | 39.213 | 41.512 | 42.346 | 44.154 | 47.701 | 48.054 | 41.810 |
| Shaanxi | 48.170 | 51.992 | 49.815 | 48.978 | 48.201 | 47.702 | 48.323 | 50.019 | 54.806 | 48.528 |
| Gansu | 52.915 | 55.043 | 57.001 | 53.642 | 50.253 | 48.673 | 43.114 | 42.063 | 45.361 | 42.422 |
| Qinghai | 28.619 | 17.706 | 19.409 | 31.359 | 24.698 | 20.764 | 25.663 | 38.723 | 38.251 | 40.669 |
| Ningxia | 63.665 | 63.158 | 62.449 | 62.513 | 61.907 | 59.416 | 57.732 | 55.293 | 61.848 | 55.590 |
| Xinjiang | 46.313 | 49.238 | 47.337 | 40.371 | 36.689 | 44.044 | 39.547 | 41.744 | 51.654 | 44.877 |
